# Effect of permeation enhancers on in vitro release and transdermal delivery of lamotrigine from Eudragit^®^RS100 polymer matrix-type drug in adhesive patches

**DOI:** 10.1007/s40204-019-0114-9

**Published:** 2019-05-08

**Authors:** Ifrah Jafri, Muhammad Harris Shoaib, Rabia Ismail Yousuf, Fatima Ramzan Ali

**Affiliations:** Department of Pharmaceutics, Faculty of Pharmacy and Pharmaceutical Sciences, University of Karachi, Karachi, Pakistan

**Keywords:** Lamotrigine, Transdermal, Permeation enhancer, Oleic acid, Propylene glycol, Eudragit^®^RS100

## Abstract

The drug-in-adhesive (DIA)-type matrix patches of lamotrigine are developed using variable permeation enhancers (oleic acid, PG, lemon oil and aloe vera), and drug in vitro release and its permeation are evaluated. Lamotrigine has been long used as an anti-epileptic, mood stabilizer, to treat bipolar disorder in adults and off label as an antidepressant. lamotrigine matrix patches comprising of Eudragit^®^RS100 (rate-controlling polymer) and DuroTak^®^ 387-2510 (adhesive) were prepared by pouring the solution on backing membrane (3M-9720). The thickness of 120 µm was adjusted through micrometer film applicator. USP Apparatus V was used for the evaluation of release profile, which was fitted into various mathematical models. Quality characteristics of patches were determined through weight variation, moisture content, moisture uptake and drug content evaluation. FTIR studies were performed for drug-excipient compatibility; Franz diffusion cell was employed for studying in vitro permeation parameters such as flux, lag time, and ER. Skin sensitivity study of optimized patch was also performed. The release from patches comprising of PG and oleic acid was maximum, i.e., 96.24 ± 1.15% and 91.12 ± 1.11%, respectively. Formulations (A1–A5) exhibited Makoid–Banakar release profile. Formulation A3 consisting of oleic acid was optimized due to enhanced permeation of drug across skin compared to other enhancers with enhancement ratio of 3.55. Skin sensitivity study revealed patch as safe and non-allergenic. The study demonstrates that oleic acid can be used as a suitable permeation enhancer for transdermal delivery of lamotrigine from matrix-type patches.

## Introduction

Transdermal drug delivery systems are suitable alternatives to conventional delivery routes and possess advantages of predicted and controlled rate of transport, avoiding first-pass effect and improved patient compliance (Prausnitz and Langer 2008). However, the barrier properties of human skin restrict the delivery of drug molecules across it. Chemical permeation enhancers by interacting with skin constituents improve the flux of drug molecules (Williams and Barry 2012). Thus, the use of chemical enhancers in transdermal preparations for enhancing the permeability of small lipophilic molecules has a long history (Karande et al. 2006).

The rate of delivery of much simpler and easy to fabricate matrix-type transdermal patches is usually controlled by skin permeation (Prausnitz et al. 2004). These systems comprise drug in the hydrophilic or lipophilic matrix (Valenta and Auner 2004). The current study focuses on developing a matrix-type transdermal patch of lamotrigine, a relatively new anti-epileptic agent which is not structurally related to compounds of this class but has pharmacological action similar to phenytoin (Leach et al. 1986). It is a weak base with pKa of 5.5 and molecular weight 256.09. The octanol/water partition coefficient is 1.19. The low water solubility of lamotrigine, i.e., 1 g/L presents a challenge in designing formulation (Levy et al. 2002; Rambeck and Wolf 1993). Lamotrigine is used alone or in combination with other anticonvulsants for treating both partial and generalized seizures. It is conventionally available as immediate-release and extended-release tablet dosage forms. The co-administration of other anti-convulsants and dosing recommendations for lamotrigine are variable and, to optimize treatment, therapeutic drug monitoring is recommended as lamotrigine linear pharmacokinetics which can be significantly affected by co-administration of other AEDs. The elimination rate of lamotrigine is increased due to enzyme-inducing drugs, such as phenytoin, carbamazepine, phenobarbital, and primidone, and prolonged by administration of valproic acid (Yuen et al. 1992; Gidal et al. 2000; Anderson et al. 2002).

Hence, the objective of this study was to develop a transdermal patch of lamotrigine for the better management of anti-convulsive therapy, alone or in combination. Transdermal patches avoid first-pass metabolism and require lower initial concentration for therapeutic effect (Hillery and Park 2016). This study also helps in assessing the impact of various permeation enhancers on in vitro release and skin permeation of drug from matrix patches. The compatibility of the drug with excipients was also studied through FTIR technique. The optimized patch was further evaluated for skin sensitivity reaction to ensure that the formulated patch is suitable for skin application.

## Materials and methods

### Materials

Lamotrigine was gifted from Hilton Pharmaceuticals Pvt. Ltd. Other chemicals/reagents/excipients included Eudragit^®^ RS 100 (Evonik, Darmstadt, Germany), Ethanol, dicholoromethane, dibutyl phthalate (DBP), aloe, oleic acid, propylene glycol (PG), lemon oil, potassium dihydrogen phosphate and sodium hydroxide (Merck, Darmstadt, Germany). The patch components such as backing membrane (3M-9720) and release liner (SCOTCHPAK 9755) were gifted by 3M, St. Paul, USA. Acrylate adhesive Duro-Tak^®^ 387/2510 was supplied by Henkel Corporation (Bridgewater, USA).

### Animals

Albino Wistar rats weighing 150–180 g were used for in vitro skin permeation and skin sensitivity studies. The animals were provided by the animal house of the Faculty of Pharmacy and Pharmaceutical Sciences, University of Karachi.

### Ethics statement

All ARRIVE guidelines were followed for the care and handling of animals. The study was approved by the Board of Advanced Studies and Research, University of Karachi.

### Preparation of matrix former

Matrix-forming polymer was prepared through a method reported by Minghetti 2007. Briefly, weighed amount of Eudragit^®^RS100 was dissolved into a mixture of ethanol and dicholormethane (8:2). The solution was then sonicated for 15 min and stored at room temperature (Minghetti 2007).

### Fabrication of matrix patch of lamotrigine

The matrix-type patches A1–A5 were prepared using various permeation enhancers as shown in Table 1. Fixed amount of drug (30 mg), adhesive (DuroTak^®^ 387-2510 10 mg), Eudragit^®^ RS100 matrix polymer mixture (4% m/m), plasticizer (DBP 1 mL), and enhancer (2 mL to numerated patch A2–A5) were mixed in ethanol (10 mL), and for control patch (A1) permeation enhancer was not added in the solution mixture. The solution was magnetically stirred for 1 h at room temperature. The solution was then spread on release liner (Scotchpak 9755) through micrometer film applicator (Sheen Instruments, UK) to obtain 120-µm-thick wet film. The patches were air dried at room temperature for 1 h and then were laminated with a backing membrane (CoTran 9720). The patches were cut to appropriate size, packed in aluminum foil and stored at room temperature.

**Table 1:** Composition of the lamotrigine matrix patches

Formulation code	Drug (lamotrigine) (mg)	Plasticizer (dibutyl phthalate) (mL)	Rate-controlling polymer (Eudragit^®^ RS100) (mg)	Adhesive (DuroTak^®^ 387-2510) (mg)	Permeation enhancers (PE)					Solvent (ethanol) (mL)
					No PE	Lemon oil (mL)	Oleic acid (mL)	PG (mL)	Aloe (mL)	
A1	30	1	40	10	–	–	–	–	–	10
A2	30	1	40	10	–	2	–	–	–	10
A3	30	1	40	10	–	–	2	–	–	10
A4	30	1	40	10	–	–	–	2	–	10
A5	30	1	40	10	–	–	–	–	2	10

### Characterization of matrix-type DIA patches

The formulated patches were characterized through weight variation, moisture content and moisture uptake by the patch. The weight of (*n* = 6) patches of each formulation was determined through analytical balance (Sartorius CP-2245, Gottingen, Germany).

Moisture content and moisture uptake were assessed according to the method reported by (Arora and Mukherjee 2002). The individually weighed patches were stored in activated silica containing desiccator for 24 h at room temperature. The patches were individually weighed again until constant weight was obtained. The moisture content was computed by comparing the difference between initial and final weights with final weight.

The weighed patches placed in a desiccator for 24 h at room temperature were exposed to relative humidity of 84% (a saturated solution of potassium chloride). The weight obtained by patches was determined and the moisture uptake was estimated by comparing the difference of final and initial weight with initial weight.

### Fourier-transform infrared spectroscopy

The interaction between drug and excipients of lamotrigine matrix patches was studied through FTIR analysis (Thermo-Nicollet AVATAR 5700, Madison, USA). For this purpose, the patch components were finely powdered and mixed with oven dried potassium bromide powder to form pellets. The sample was then scanned at wide spectral range of 7800–375 cm^−1^. The spectrum was analyzed through EZ OMNIC^®^ version 7.

### Drug content

The content of lamotrigine from the formulated patches was estimated through a method reported by (Prajapati et al. 2011). The strip of patches (*n* = 6) having surface area of (1 cm^2^) was cut and dissolved in phosphate buffer saline pH 7.4 through sonication for 15 min. The solution was then stirred magnetically for 24 h. The resultant solution was filtered (Whatman filter paper) and analyzed spectrophotometrically at 267 nm (UV-1800, Shimadzu Corporation Kyoto, Japan). The absorbance of sample was compared with that of standard at concentration of 40 µg/mL.

### In vitro release study

The release of lamotrigine from matrix patches (*n* = 5) comprising of different permeation enhancers was determined through USP apparatus 5, paddle over disk (Erweka DT-600, Heusenstamm. Germany). A 900 mL of phosphate buffer pH 7.4 was placed in the vessel. Temperature was adjusted to 32 ± 0.2 °C. The patch was placed on USP transdermal sandwich (90 mm diameter, 17″ mesh) (Labecx, Santa Clarita California, USA) and immersed into the medium such that the patch was placed flat. The rotation of paddle was adjusted at 50 rpm. Aliquots of 10 mL were drawn and replaced with fresh medium at specified time points, i.e., 15 and 30 min, and 1, 2, 3, 4, 6, 8 and 12 h. The samples were diluted and analyzed through UV spectrophotometer (UV-1800, Shimadzu Corporation Kyoto, Japan) at 267 nm. Mathematical models such as zero order, first order, Higuchi, Korsmeyer–Peppas, Weibull and Makoid–Banakar (Eqs. 1–6) were applied to quantify the results (Costa and Sousa Lobo 2003) The coefficient of correlation and rate constants were determined using DDSolver^®^ (Zhang et al. 2010):

1 $$Q_{t} = Q_{o} + k_{0} t,$$

2 $$\log Q_{t} = \log Q_{o} + \frac{{k_{1} t}}{2.303},$$

3 $$Q_{t} = K_{H} t^{1/2} ,$$

4 $$Q_{t} = at^{n} ,$$

5 $$m = 1 - { \exp }\left[ {\frac{{ - \left( {t - T_{l} } \right)^{b} }}{a}} \right],$$

6 $$\frac{{M_{t} }}{{M_{\infty } }} = k_{\text{MB}} t^{n} e^{{\left( { - {\text{ct}}} \right)}} ,$$

where *Q*_*o*_ and *Q*_*t*_ are the initial amount of drug in dosage form and drug released at time *t*, respectively. *k*_o_, *k*_1_ and *k*_H_ are zero-order, first-order and Higuchi rate constants. In Korsmeyer–Peppas model (Eq. 4), “*a*” is structural and geometric characteristic of dosage form and “*n*” is release exponent. In Eq. 5, “*m*” represents the drug accumulation at time “*t*”, “*a*” and “*b*” are scale and shape parameter, respectively, and lag time is represented by “*T*_l_“. “*k*_MB_”, “*n*” and “*c*” are empirical parameters and “*M*_t_/*M*_∞_” is accumulation fraction of drug at time “*t*” (Eq. 6).

### In vitro permeation study

The permeation study was performed in a Franz diffusion cell (Orchid Scientific and Innovative, India). Freshly excised rat skin was soaked in normal saline; the excess fats and tissues were removed. The skin was then immersed in phosphate buffer saline (PBS) pH 7.4 prior to experiment and was cut into required size.

The receptor compartment was filled with 5 mL phosphate buffer saline (PBS), pH 7.4, and the temperature of the assembly was maintained at 32 ± 0.2 °C through water circulating jacket. The receptor compartment was magnetic stirred at 450 rpm. The patches were fixed securely on the skin so that hairless epidermis (stratum corneum) was located between the donor and receptor compartments. The surface area of both skin and patch was 1.78 cm^2^. The experiment for each formulation was repeated four times.

The sample of 0.6 mL was drawn at specified time points and was replaced with an equal volume of medium to maintain the sink condition. The samples were analyzed spectrophotometrically at 267 nm. The permeation parameters such as flux, time lag, permeability coefficient (Eq. 7), diffusion coefficient (Eq. 8), enhancement ratio (Eq. 9), best-fit equation and regression coefficient were determined according to the method stated by (Ubaidulla et al. 2007). Flux and time lag are determined by slope and x-intercept of the linear plot between the amount permeated and time, respectively. The fraction of rate controlled by device and skin was also calculated (Eqs. 10, 11) (Kalia and Guy 2001),

7 $$P = \frac{J}{C},$$

8 $$DC = \frac{{h^{2} }}{6L},$$

9 $$ER = \frac{{J_{\text{PE}} }}{{J_{\text{control}} }},$$

10 $$F_{\text{D}} = \frac{{M_{\text{total}} }}{{M_{\text{device}} }},$$

11 $$F_{\text{s}} = 1 - F_{\text{D}} ,$$

where *P* and DC are the permeation and diffusion coefficients, respectively. “*J*”, “*J*_PE_” and “*J*_control_” are the flux, flux of patch containing permeation enhancer and flux of patch without permeation enhancer, respectively. “*C*” is the concentration of drug in a patch, “*h*” is thickness of skin membrane and “*L*” is the time lag. “*F*_D_” and “*F*_s_” are the fraction rates controlled by device and skin while “*M*_total_” and “*M*_device_” are the total permeation of drug from skin and total release of drug from device, respectively.

### Skin sensitivity reaction

Skin sensitivity studies were performed as reported by (Wang et al. 2008). The Albino Wistar rats were divided into three groups (*n* = 5). The animals were kept in laboratory for 7 days before test to acclimatize with the environment. Dorsal abdominal skin of rat was shaved using electric clipper 1 day before experiment. No treatment was given to the control group, i.e., group 1. The lamotrigine patch having maximum flux was selected for skin sensitivity study that was applied on group 2 animals. The standard irritant 0.8% v/v formalin solution was applied on the skin of animals of group 3 to compare the effects. The signs of irritation and erythema were visually observed for 6 days and were scored according to the method mentioned by Draize et al. (Draize et al. 1944).

## Result and discussion

### Characterization of lamotrigine matrix-type DIA patches

The lamotrigine patches were characterized, and results are shown in Table 2. The mean weight of formulations A1–A5 ranged from 960 ± 9.54 mg to 970 ± 4.47 mg and the percent drug loading was found between 95.5 ± 0.4 and 98.2 ± 0.9%. The results specify that weight of different formulations was approximately similar with drug loading within specified limits of 90–110%. This similarity in weight and uniformity in drug content indicate that the method of patch preparation was efficient enough to produce patches with minimum variability in drug content and can be employed in preparing matrix patches of lamotrigine at commercial scale.

**Table 2:** Characterization of the lamotrigine matrix patches (A1–A5)

Formulation code	Mean ± SD (*n* = 6)			
	Weight (mg)	Moisture content (%)	Moisture uptake (%)	Drug content (%)
A1	970 ± 4.47	0.52 ± 0.022	0.72 ± 0.014	95.5 ± 0.4
A2	964 ± 6.35	0.21 ± 0.014	0.31 ± 0.020	97.5 ± 1.1
A3	960 ± 9.54	0.21 ± 0.017	0.21 ± 0.026	97.7 ± 1.3
A4	962 ± 9.36	0.10 ± 0.020	0.42 ± 0.017	97.8 ± 1.4
A5	966 ± 8.91	0.10 ± 0.023	0.52 ± 0.019	98.2 ± 0.9

The moisture content of the matrix patches must be low so that the patches remain stable and do not completely dry to form brittle patches (Gannu et al. 2007). The small moisture content ranging from 0.10 ± 0.23 to 0.52 ± 0.022 indicates that a stable formulation was prepared. The moisture uptake by patches was also less, i.e., between 0.21 ± 0.026 and 0.72 ± 0.014%. This is important for preventing microbial contamination and also lowers bulkiness (Ubaidulla et al. 2007).

### Fourier-transform infrared spectroscopy

**Fig. 1:**
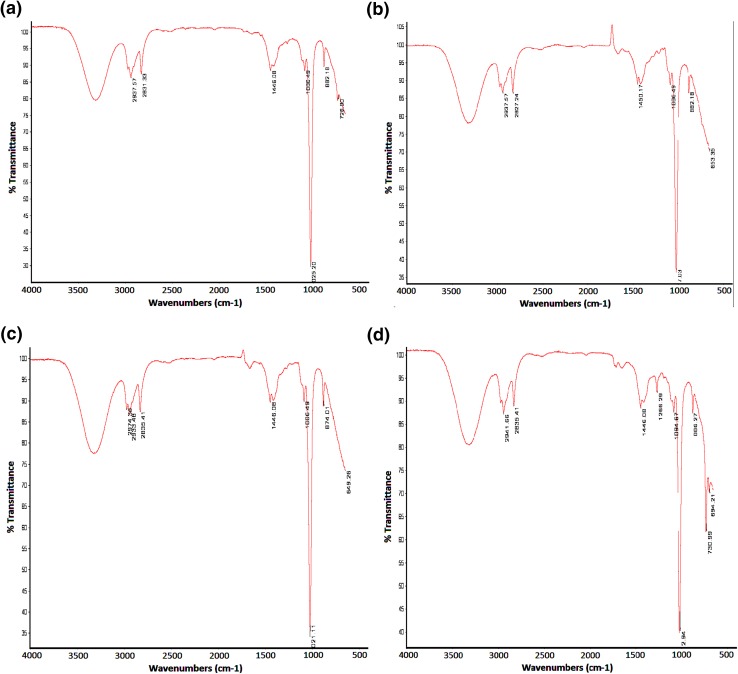
FTIR spectral formulations **a** A2, **b** A3, **c** A4 and **d** A5

Figure 1 represents the FTIR spectra of patches consisting of variable permeation enhancers. The characteristic C–Cl peak of lamotrigine was observed between 600 and 800 cm^−1^, the peak at 1006.49 cm^−1^ indicated C–H bend, and –CH alkyl stretch was observed at 2936.57 cm^−1^ in Fig. 1a and b, at 2933.48 cm^−1^ in c and at 2941.66 cm^−1^ in d. All peaks in every formulation were found within similar ranges except in Fig. 1c, and a peak at 2974.36 cm^−1^ was observed also representing CH stretch. No overlapping or merging of spectral peak was observed indicating that there is no interaction between polymer, enhancer and drug. The FTIR drug-excipient compatibility study of matrix-type transdermal patches is extensively reported in the literature (Mukherjee et al. 2005; Prasad Verma and Chandak 2009; Rajabalaya 2010; Rajan et al. 2009; Shinde et al. 2008; Ubaidulla et al. 2007). Eudragit RS used as matrix-forming agent is found compatible with most of the drugs (Wade and Weller 1994).

### In vitro release study

**Fig. 2:**
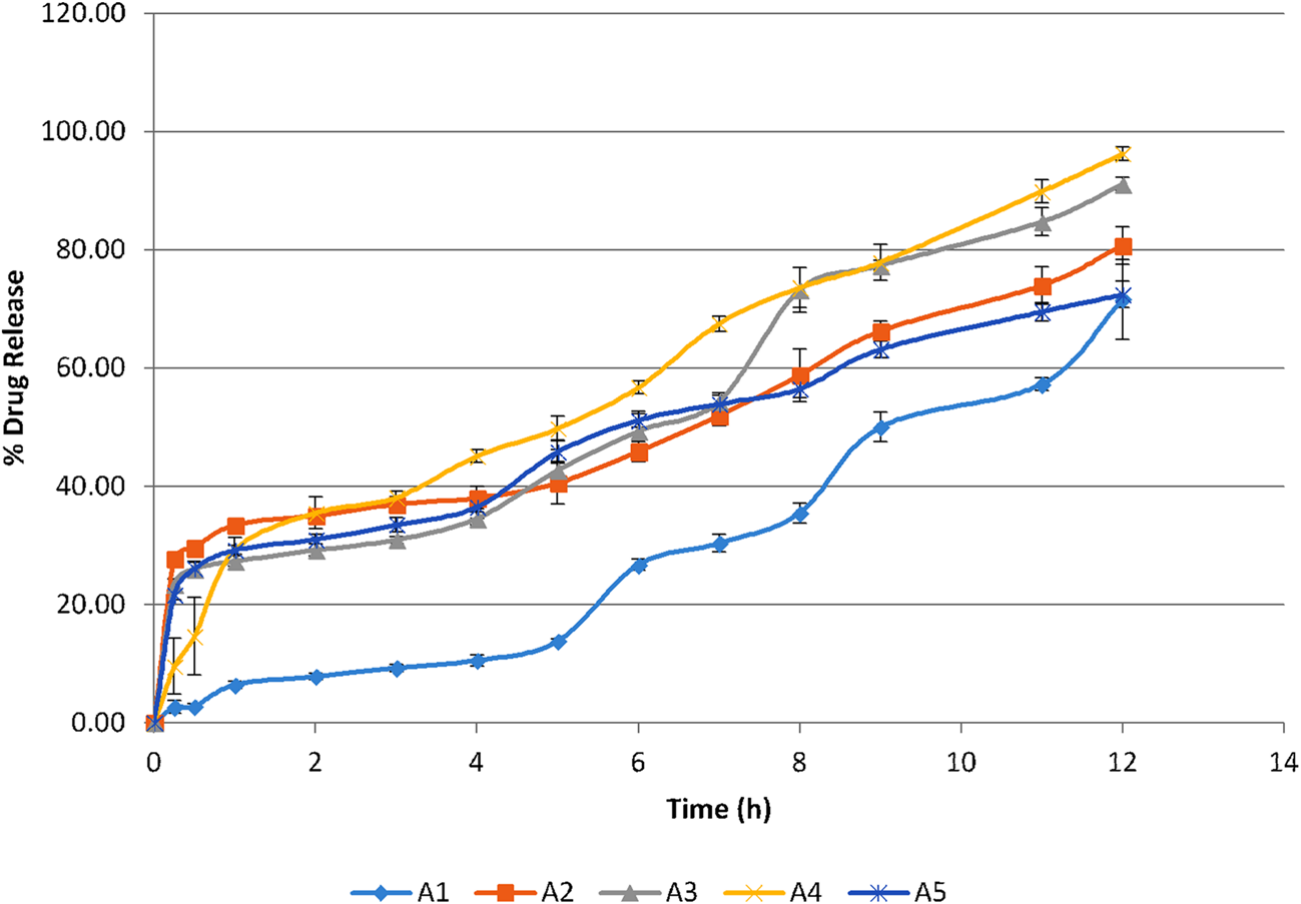
In vitro release profile of lamotrigine matrix patches (A1–A5)

Drug release as demonstrated in Fig. 2 in 12 h is the maximum for formulations A3 and A4, that is, 91.12 ± 1.11% and 96.24 ± 1.15% containing oleic acid and propylene glycol as permeation enhancers, respectively. Propylene glycol possibly by forming hydrophilic micropores in the adhesive layer increases the release of drug from matrix patches. This was also comparable to the findings of Guyot and Fawaz in which the release of propranolol was increased due to the presence of propylene glycol (Guyot and Fawaz 2000). Eudragit^®^ polymers exhibit sustained release features providing a rate-controlling membranes for transdermal systems and different categories of permeation enhancers possessing different characteristics which were used to assess the release from matrix polymer by balancing the hydrophobic character of matrix former. (Misra 2014). In control formulation (A1), the initial burst effect and final excessive release were not observed indicating that the system comprising of hydrophobic polymer and the adhesive exhibits controlled release mechanism. However, the releases can be controlled by the addition and elimination of different quantities of adhesive, hydrophobic matrix and chemical enhancer, and thereby modifying according to the formulation type. Similar studies were conducted by Aggarwal where the matrix formulation consisting of Eudragit^®^ RS showed a controlled release behavior (Aggarwal et al. 2013).

Whereas formulation A2 showed an optimum release of 77% at the end of the experiment, which is evident as per studies conducted by Jiang that essential oils such as terpenes which are generally regarded as safe (GRAS) being hydrophobic in nature take a bit longer t to interact with the buffer medium and drug dissolution thus, a sustained effect is obtained and by altering the concentration of the oil the release of the drug can be regulated. (Jiang et al. 2017).

Formulation A5, where aloe vera was added considering it to be as a friendly skin enhancer, a release of just 70% was observed indicating that despite of being used as a supportive vehicle in many studies, aloe did not exceptionally produce an effect with respect to lamotrigine.

All formulations as specified in Table 3 exhibited Makoid–Banakar release profile. When the c value of Makoid–Banakar model approaches zero, the model becomes identical to Korsmeyer–Peppas model (Al-Achi et al. 2013). The release exponent ‘n’ indicates the type of diffusion followed by the formulation. In the current study, n values of formulation A3 and A4 were found between 0.5 and 1 showing Non-Fickian transport (Costa and Lobo 2001). Estradiol matrix patches were best fitted to Higuchi and Korsmeyer–Peppas and Makoid–Banakar release models. The value of n for Formulation A3 and A4 was between 0.5 and 1 indicating an anomalous transport, while A2 and A5 exhibited Fickian diffusion. The shape parameter β indicated that A1 and A3 have sigmoid S-shaped curve with value more than 1 while A2, A4 and A5 values are less than 1 with initial higher slope and parabolic shape curve. Shape parameter of less than 1 was observed in the case of estradiol patch (Costa and Lobo 2001; Costa and Sousa Lobo 2003). Verapamil matrix patches exhibited zero order and Higuchi release mechanism (Kusum Devi et al. 2003).

**Table 3:** Model fitting of lamotrigine matrix patches (A1–A5) release profile

Mathematical models	A1	A2	A3	A4	A5
Zero order					
*R*^2^	0.9701	0.9422	0.9701	0.9821	0.9558
*k*_o_ (h^−1^)	5.017	7.414	8.240	8.906	7.133
First order					
*R*^2^	0.9431	0.9230	0.9385	0.9797	0.9626
*k*_1_ (h^−1^)	0.063	0.125	0.143	0.170	0.119
Higuchi model					
*R*^2^	0.8934	0.9448	0.9472	0.9896	0.9783
*k*_H_ (h^−1/2^)	13.435	21.655	23.535	25.505	20.876
Korsmeyer–Peppas					
*R*^2^	0.9915	0.9404	0.9560	0.9932	0.9770
*N*	1.524	0.365	0.599	0.600	0.392
*K*_kp_ (h^−n^)	1.584	28.065	19.345	20.947	25.730
Weibull					
*R*^2^	0.9923	0.9398	0.9707	0.9932	0.9767
*A*	1282.570	794,556.783	161.762	2,251,097.401	90.271
*B*	1.975	0.658	1.368	0.603	0.398
Makoid–Banakar					
*R*^2^	0.9926	0.9959	0.9855	0.9969	0.9944
*N*	2.937	-0.002	0.042	0.312	0.156
*k*_MB_	0.058	28.092	25.195	25.753	26.470
*C*	0.066	-0.089	-0.105	-0.048	-0.054

### In vitro skin permeation

**Fig. 3:**
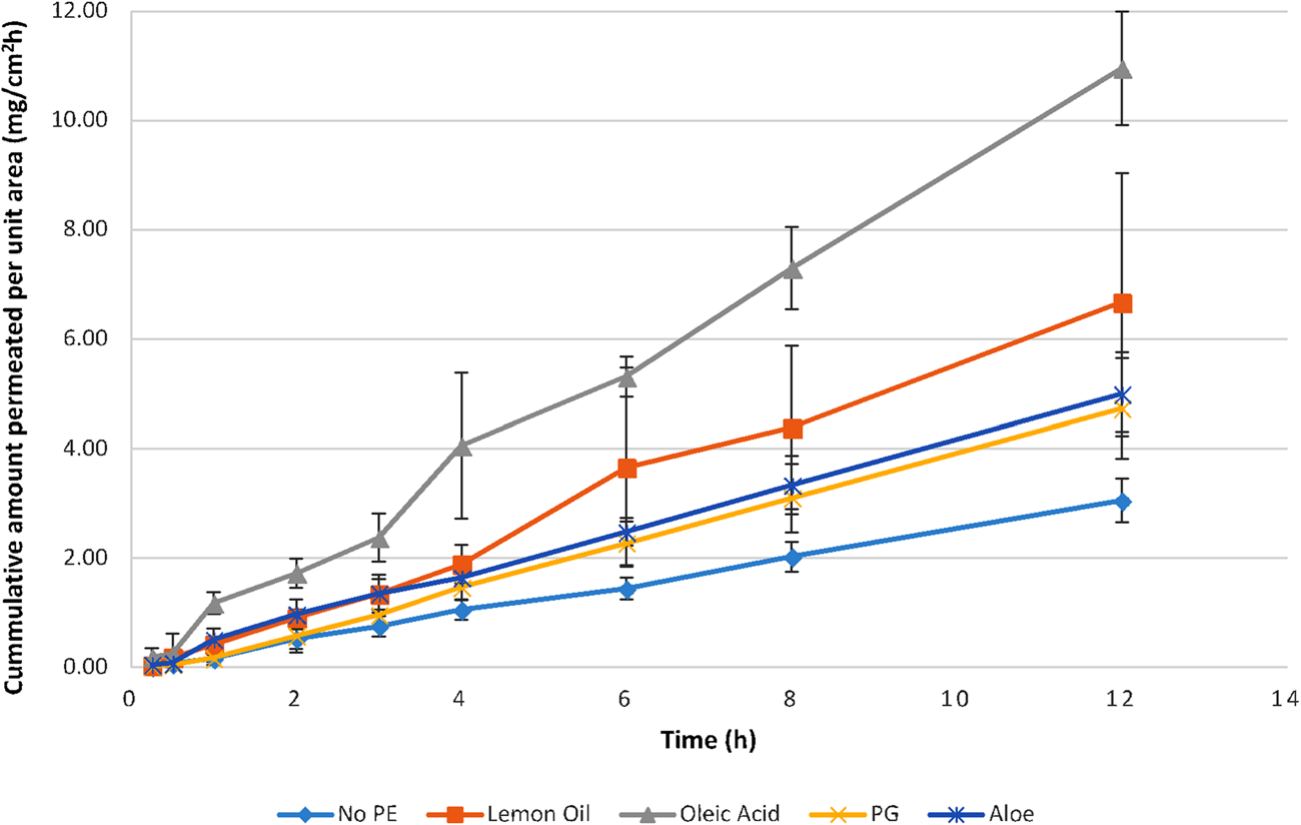
In vitro permeation profile of lamotrigine matrix patches (A1–A5)

The permeation of lamotrigine from matrix patch using various permeation enhancers was studied and specified in Fig. 3. Formulation A3 containing oleic acid showed maximum permeation at hour 12 with the highest flux of 0.916 mg/cm^2^h, enhancement ratio of 3.55, the regression coefficient of 0.9958 and best-fit equation *Q*_t_ = 0.916t–0.044. In a similar study, where Eudragit RS 100 and 10% oleic acid were used the highest permeation of drug salbutamol sulfate was observed (Misra 2014). Formulation A2 comprising of lemon oil as a penetration enhancer showed its maximum burst at 15 min that might be because some essential oils are compatible with the buffer medium that means they would not take longer to solubilize inside a solvent thus letting drug to be exposed to the medium. However, the polymer, Eudragit^®^ RS resisted the buffer exposure and controlled the release by 77% at the hour 12. Similar studies were conducted by Jiang et al., where different permeation enhancers were used and concluded that essential oils are safe, non-toxic and an effective way of delivering many drugs across skin by causing a reversible change in the stratum corneum. (Jiang et al. 2017).

Permeation parameters for formulations are presented in Table 4. Formulation A3 consisting of oleic acid as enhancer exhibited maximum permeability with ER of 3.55. Oleic acid mainly affects the lipids of stratum corneum; its uptake by stratum corneum decreases the transition temperature of lipid and enhances the permeation of drug (Francoeur et al. 1990). It also acts by decreasing the viscosity of lipids of superficial layer (Naik et al. 1995). Touitou et al. studied the effect of various penetration enhancers on morphology of epidermal Langerhans cell and concluded that oleic acid has prominent effect on skin morphology hence improves permeation across skin (Touitou et al. 2002).

**Table 4:** Permeation Parameters of (A1–A5) lamotrigine matrix patches

Permeation parameters	A1	A2	A3	A4	A5
Flux (mg/cm^2^h)	0.258	0.575	0.916	0.409	0.416
Intercept	− 0.054	− 0.188	− 0.044	− 0.193	− 0.005
*R* ^2^	0.9982	0.9935	0.9958	0.9987	0.9973
Permeation coefficient (P) (cm/h)	0.009	0.019	0.031	0.014	0.014
Time lag (L) (min)	12.6	19.8	3	1.41	0.6
Diffusion coefficient (DC) (cm^2^/h)	2.35E−04	1.51E−04	1.02E−03	1.05E−04	3.78E−03
Enhancement ratio (ER)	1.00	2.22	3.55	1.58	1.61
*F*_D_	0.252	0.490	0.713	0.292	0.409
*F*_s_	0.748	0.510	0.287	0.708	0.591
Best-fit equation	*Q*_t_ = 0.258t-0.054	*Q*_t_ = 0.575t-0.188	*Q*_t_ = 0.916t-0.044	*Q*_t_ = 0.409t-0.193	*Q*_t_ = 0.416t-0.005

The fraction rate controlled by device is found between 0.252 and 0.713 indicating that the rate is not controlled by device and influence of permeation parameters is involved (Kalia and Guy 2001).

### Skin sensitivity reaction

**Fig. 4:**
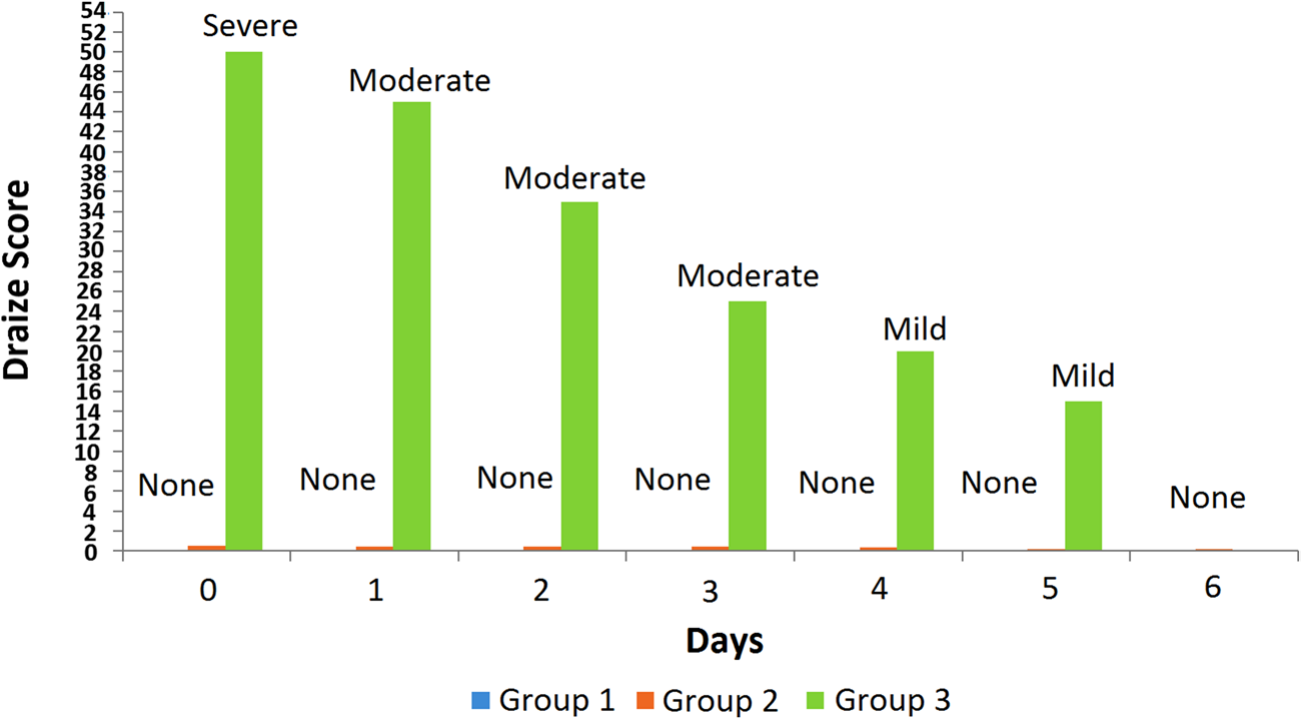
Graphical representation of skin sensitivity reaction through Draize score

The skin irritation causes slight pain and discomfort at the area of application and in 1.7–6.8% cases patches are discontinued due to skin reactions. The skin sensitivity reactions caused by patches are mostly localized and resolve after a few days of patch removal. The rotation of the application site can also minimize the chances of such reaction (Ale et al. 2009). The pressure-sensitive adhesive used in transdermal preparation may lead to skin irritation reaction and must be evaluated prior to application on the skin (Venkatraman and Gale 1998). Skin sensitivity reaction of formulation A3 was studied and compared with the standard irritant. The skin condition was visually inspected for irritation, allergy, redness and rashes, and scored according to Draize method (Fig. 4). It was observed that animals of group 3 showed severe skin reaction on the day of application and moderate for the next 3 days followed by a mild and then normal skin condition. The test group (group 2) exhibited no skin reaction, which was comparable to the skin condition of control group 1 animals indicating the formulated patch does not cause any skin sensitivity reaction.

## Conclusion

The lamotrigine matrix-type DIA transdermal patches comprising of different permeation enhancers were formulated. Release exhibited suitable attributes fitting best to Makoid–Banakar-order kinetics. The drug was also found compatible with the excipients. Patch adhered to the surfaces in a compliant manner without causing apparent damages. All formulations showed good physical stability. Since formulation A3 containing oleic acid revealed a good in vitro release and permeation of drug, patch A3 was known to be best suited formulation amongst all for delivery of drug (lamotrigine) across the skin safely without causing even minor harm to the skin and thus can be possibly used as an alternative delivery route for administration of lamotrigine.
